# Analysis to Estimate Genetic Variations in the Idarubicin-Resistant Derivative MOLT-3

**DOI:** 10.3390/ijms18010012

**Published:** 2016-12-22

**Authors:** Tomoyoshi Komiyama, Atsushi Ogura, Takatsugu Hirokawa, Miao Zhijing, Hiroshi Kamiguchi, Satomi Asai, Hayato Miyachi, Hiroyuki Kobayashi

**Affiliations:** 1 Department of Clinical Pharmacology, Tokai University School of Medicine, 143 Shimokasuya, Isehara, Kanagawa 259-1193, Japan; mrprince329@yahoo.co.jp; 2 Nagahama Institute of Bio-Science and Technology, Nagahama, Shiga 526-0829, Japan; aogu@whelix.info; 3 The National Institute of Advanced Industrial Science and Technology (AIST), Tokyo Waterfront Bio-IT Research Building 2-4-7 Aomi, Koto-ku, Tokyo 135-0064, Japan; t-hirokawa@aist.go.jp; 4 Support Center for Medical Research and Education, Tokai University, 143 Shimokasuya, Isehara, Kanagawa 259-1193, Japan; kamiguti@is.icc.u-tokai.ac.jp; 5 Department of Laboratory Medicine, Tokai University School of Medicine, 143 Shimokasuya, Isehara, Kanagawa 259-1193, Japan; sa@is.icc.u-tokai.ac.jp (S.A.); miyachi@is.icc.u-tokai.ac.jp (H.M.)

**Keywords:** leukemia, MOLT-3, *GALNT2* gene, gene polymorphism, mitochondrial DNA, CGH array, genetic variations, idarubicin (IDR)

## Abstract

Gene alterations are a well-established mechanism leading to drug resistance in acute leukemia cells. A full understanding of the mechanisms of drug resistance in these cells will facilitate more effective chemotherapy. In this study, we investigated the mechanism(s) of drug resistance in the human acute leukemia cell line MOLT-3 and its idarubicin-resistant derivative MOLT-3/IDR through complete mitochondrial and nuclear DNA analyses. We identified genetic differences between these two cell lines. The ND3 mutation site (p.Thr61Ile) in the mitochondrial DNA sequence was unique to MOLT-3/IDR cells. Moreover, we identified five candidate genes harboring genetic alterations, including *GALNT2*, via CGH array analysis. Sequencing of the *GALNT2* exon revealed a G1716K mutation present within the stop codon in MOLT-3/IDR cells but absent from MOLT-3 cells. This mutation led to an additional 18 amino acids in the protein encoded by *GALNT2*. Using real-time PCR, we determined an expression value for this gene of 0.35. Protein structure predictions confirmed a structural change in *GALNT2* in MOLT-3/IDR cells that corresponded to the site of the mutation. We speculate that this mutation may be related to idarubicin resistance.

## 1. Introduction

The Ministry of Health, Labour, and Welfare of Japan reports that the annual numbers of deaths attributable to leukemia in Japan are approximately 5000 among men and 3000 among women [1]. Despite these high mortality rates, the pathogenesis of leukemia is still not as well understood as that of other cancers. Furthermore, in recent years, the leukemia mortality rate has decreased gradually as a result of advances in treatment such as bone marrow transplantation, the development of anti-infective agents, optimization of transfusion therapy, evaluation of treatment protocols through multicenter studies, and development of molecular targeted drugs [2].

Leukemia patients often respond differently to treatment, and effective treatments have not been clearly established, particularly for relapsed disease. This limitation is attributed partly to the resistance of leukemia cells to anti-leukemic drugs. In general, agents used to treat leukemia have been based on the “total cell kill theory”, as cytotoxic anti-leukemic drugs cannot discriminate abnormal cells from normal cells. Usually, several anti-leukemic drugs are used in the context of multidrug therapy [3]; for example, patients with acute leukemia often receive daunorubicin or idarubicin (IDR) for three days and cytarabine (standard dose) for seven days [4]. However, the combination of these standard therapies with other cytotoxic agents is not recommended because of the risk of adverse reactions such as fever, rash, anorexia, nausea, dyspnea, and cardiac events [5].

Acute leukemia accounts for approximately 80% of acute leukemia cases in Japanese adult patients [1]. Among the available treatments [2], idarubicin has been used to treat refractory and relapsed acute lymphoblastic leukemia [6,7,8,9,10,11]. The anti-tumor effects of IDR are mediated through the inhibition of DNA transcription to RNA and activation of the aryl hydrocarbon receptor (AhR). The AhR is a ligand-activated transcription factor involved in the regulation of biological responses to planar aromatic (aryl) hydrocarbons [12,13]. Benzene-induced leukemogenesis is thought to involve various pathways and biological processes such as AhR dysregulation, as well as apoptosis, proliferation, differentiation, oxidative stress, and reduced immunosurveillance [14,15].

IDR is highly lipophilic, thus allowing the maintenance of a high intracellular drug concentration even in P-glycoprotein expressing cells [16,17]. However, drug resistance to IDR is an increasing problem and is proving to be a significant barrier to treatment. Previous studies have demonstrated aberrant intracellular signal activation in a variety of malignant cells. These aberrant signals correlate closely with anti-leukemic drug resistance mechanisms and the progression of malignancy. Under normal conditions, signaling pathways control organ size, tissue regeneration, and stem cell renewal. However, dysregulation of these signaling pathways affects tumorigenesis and cancer metastasis, as well as drug resistance [18,19]. Various biomolecules related to drug resistance have played important roles in the proliferation and survival of normal cells. Generally, it may be harmful to inhibit such biomolecules because such inhibition would not discriminate between cancer cells and normal cells. One strategy, therefore, is the identification of resistance genes, or genes that are strongly expressed in abnormal cells that are resistant to a specific drug. To investigate the mechanism of resistance to idarubicin, we generated an IDR-resistant MOLT-3 cell line, designated as MOLT-3/IDR. This MOLT-3 cell line was established from the peripheral blood of a patient with relapsed acute lymphoblastic leukemia following treatment with multidrug chemotherapy. The cell line originated from the leukemic T lymphocytes [20].

In this study, we subjected IDR-resistant leukemia cells (MOLT-3/IDR) to gene expression profiling via cDNA microarray. In our previous study, we confirmed the decreased expression of topoisomerase IIα and increased expression of GS3955 protein and lymphocyte-specific protein tyrosine kinase in this MOLT-3/IDR cell line (unpublished data). However, we were unable to inhibit IDR resistance by regulating the expression of these proteins alone (unpublished data), indicating the existence of some other resistance mechanism(s) in MOLT-3/IDR cells. In this study, we investigated the molecular mechanisms of IDR resistance using a human T cell leukemia line (MOLT-3) and IDR-resistant derivative line (MOLT-3/IDR). We searched for genes specifically mutated in MOLT-3/IDR cells using both mitochondrial (mtDNA) and nuclear DNA analyses. Mitochondria have previously been implicated in resistance to other drugs. Most antineoplastic agents exert anti-tumor effects by controlling mitochondrial apoptosis, and genetic mutations in tumor cell mtDNA have therefore been reported to confer resistance to chemotherapy [21,22,23,24,25]. Our findings reveal genetic alterations in leukemia cells that may be related to IDR resistance. New insights into drug resistance mechanisms in leukemia cells may lead to more effective therapeutic strategies in the future.

## 2. Results

### 2.1. Identification of Gene Polymorphisms in the Mitochondrial DNA Sequence of MOLT-3/IDR Cells

To identify gene polymorphisms in mtDNA sequences from the human T cell leukemia line (MOLT-3) [20,26] and an IDR-resistant derivative line (MOLT-3/IDR), we aligned and compared the corresponding sequences (GenBank accession numbers: LC050519 and LC050520, respectively). We identified a T7966C substitution in a *COX2* exon and C10241Y substitution (C or T; hetero site) in the *ND3* exon. We also observed a cytosine (CC) insertion at position 16,193 of the D-loop region (Table 1). Next, we focused on non-synonymous sites in these exon regions. We observed the following non-synonymous substitutions: Phe127Ser in *COX2* and Ile39Val in *ND5* of MOLT-3 cells. In MOLT-3/IDR cells, we observed non-synonymous substitutions of Thr61Ile in *ND3* and at multiple sites in *ND5*. However, we did not observe the non-synonymous Phe127Ser substitution in the *COX2* protein of MOLT-3/IDR cells. A BLAST analysis of the amino acid at position 61 of the ND3 sequence revealed that threonine (Thr) was invariably present. We therefore concluded that the amino acid substitution at position 61 of the ND3 protein sequence in MOLT-3/IDR cells was a unique mutation.

### 2.2. Identification of Candidate Genes Involved in IDR Resistance via CGH Array Analysis

We next investigated genetic mutations in MOLT-3/IDR cells using a CGH array (2 × 400 K) and the MOLT-3 parental cell line as a reference. We extracted 674 DNA regions from MOLT-3/IDR cells with signal strengths two times higher than the corresponding parental cell regions (*p*-value < 0.05; Table 2 and Table S1); nine of these regions had a signal intensity four times higher than that of parental cells (*p*-value < 0.05). By contrast, we extracted 724 regions of DNA from MOLT-3 parental cells with signal intensities two times higher than those of MOLT-3/IDR cells (*p*-value < 0.05), and one of these regions had a signal intensity four times higher than that of MOLT-3/IDR cells (*p*-value < 0.05). Furthermore, we confirmed the locations of the 10 mutated regions using the NCBI and Ensemble databases and identified five candidate genes: *GALNT2*, *DCHS2*, *ENTPD8*, *PNPLA7*, and *FBXW7*.

### 2.3. Polymorphism Analysis of Amino Acids in the GALNT2 Protein

Next, we confirmed each mutation site via exome analysis of the five genes. We decided to investigate *GALNT2* in greater depth because of its previously established role in carcinogenesis. From an exome analysis, we identified three synonymous sites, G858A (threonine), C1014Y (isoleucine), and A1197R (alanine), and a G1716K heterologous mutation within the stop codon of MOLT-3/IDR cells (Accession no: LC043141; Table 3). The heterologous mutation site G1716K (Y: tyrosine) was also located within the stop codon in MOLT-3/IDR cells (Table 3 and Figure S1), but not in MOLT-3 cells (Accession no: LC043140). This mutation led to an additional 18 amino acids in the translated *GALNT2* protein.

### 2.4. Quantitative Expression Analysis of GALNT2 Using Real-Time PCR and Reverse Transcription (RT)-PCR

**Figure 1:**
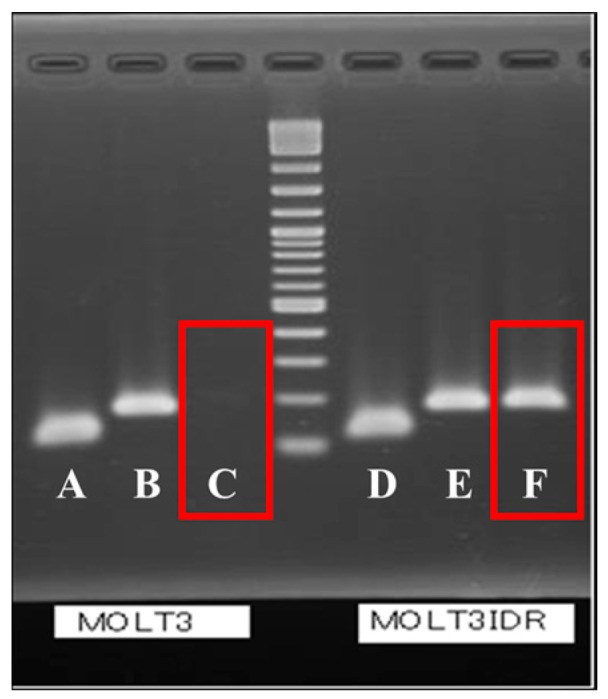
Identification of wild-type and mutant *GALNT2* in MOLT-3 and MOLT-3/IDR cells via real-time PCR. The image shows electrophoretically separated real-time PCR products amplified with the following primers: A and D, β actin (116 bp); B and E: GALNT2-wild_F + GALNT2-Rev (164 bp), C and F: GALNT2-mutant_F + GALNT2-Rev (165 bp) (Table S3).

**Figure 2:**
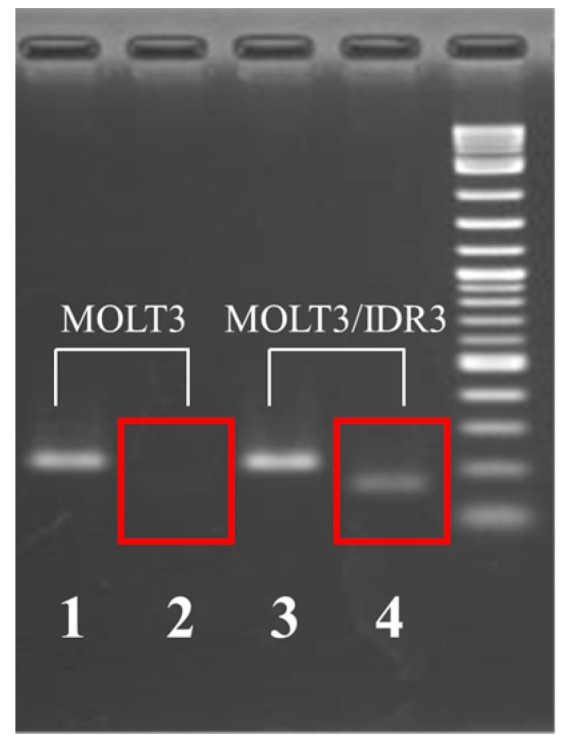
Identification of wild-type and mutant *GALNT2* in MOLT-3 and MOLT-3/IDR cells by reverse transcription (RT)-PCR. The image shows electrophoretically separated RT-PCR products. For lanes 1–4, reactions were performed at 69 °C. The following primers were used: lanes 1 and 3: GALNT2-L1 + GALNT2-Rev (215 bp); lanes 2 and 4; GALNT2-L2 + GALNT2-Rev (165 bp) (Table S3).

We investigated gene expression at the location of the stop codon by substituting K (Keto) in MOLT-3/IDR cells and performing real-time PCR and reverse transcription (RT)-PCR. PCR products were confirmed by performing real-time PCR and RT-PCR in mutated *GALNT2* (Figure 1, red rectangle and Figure S2). Moreover, the *GALNT2* (wild-type) expression level in MOLT-3 parental cells was set at 1.00 (PCR efficiency: 99.8%), and the calculated expression in MOLT-3/IDR cells was 0.96 (PCR efficiency: 99.7%). Furthermore, the mutant form of *GALNT2* was not detected in MOLT-3 wild-type cells (Figure 1 and Figure 2, red rectangles). The expression level ratio in IDR-resistant cells was 0.35 (PCR efficiency: 100%; Table 4 and Table S2).

### 2.5. Predicted Protein Structure of Mutated GALNT2

**Figure 3:**
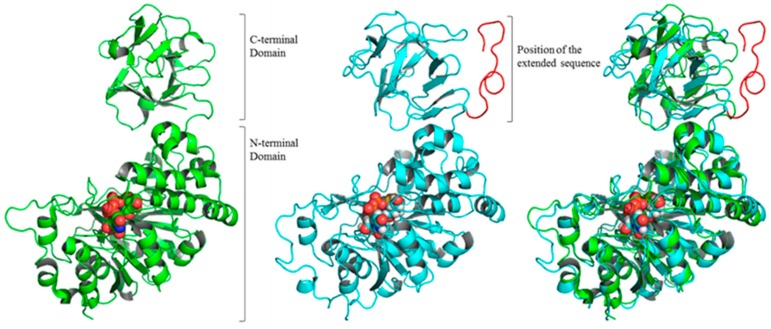
Predicted structures of wild-type and mutant *GALNT2*. The image depicts the X-ray structure [27] of wild-type *GALNT2* (**left**), predicted structure of mutated *GALNT2* (**center**), and an overlay of the structures of wild-type and mutated *GALNT2* (**right**). The extended sequence region is shown in red. The UDP-GalNAc molecule is represented by the CPK model. The structural alignment and visualization were created using PyMOL (Schrödinger LLC).

To test whether the additional 18 amino acids in mutated *GALNT2*-expressing MOLT-3/IDR cells would affect the structure of the *GALNT2* protein, we employed molecular modeling techniques. A comparison of the X-ray structure of wild-type *GALNT2* with the predicted structure of mutant *GALNT2* revealed a change in the relative positions of the N- and C-terminal domains (Figure 3 and Figure S3). The extended sequence in the mutated *GALNT2* protein appears to alter the flexibility of the hinge region located between the N- and C-terminal domains (Figure 3 and Figure S3).

## 3. Discussion

Acute leukemia is characterized by the rapid growth and accumulation of abnormal white blood cells in the bone marrow; these cells consequently interfere with the production of normal blood cells. First-line treatments for acute leukemia primarily comprise chemotherapy; however, drug resistance to anticancer agents is a major obstacle faced during leukemia therapy. The role of gene alterations in many cases of drug resistance has been well established. Identification of the mechanisms of drug resistance in acute leukemia would facilitate the development of effective chemotherapeutic strategies.

To analyze the mechanism(s) of IDR resistance, the human acute leukemia cell line MOLT-3 and its IDR-resistant derivative, MOLT-3/IDR, were used for a complete mitochondrial genome sequence analysis and CGH array analysis. Taken together, these two data sets provided us with a more accurate prediction of candidate resistance genes.

In recent years, mutations in mtDNA have been linked with several types of cancer, including bladder, breast, head and neck, lung, liver, and stomach cancers, as well as leukemia [28,29,30,31]. Furthermore, mtDNA mutations, together with reactive oxygen species (ROS), have been shown to induce the metastatic potential of carcinoma cells [32]. Increased levels of ROS induce changes in encoded respiratory chain complexes, leading to the formation of excess superoxides and consequently increasing the mutation rates of both nuclear DNA and mtDNA [25,33,34,35]. Interestingly, ROS activate nuclear factor-κB (NF-κB), a key transcription factor involved in acute and/or chronic inflammatory responses, cell proliferation, and apoptosis. NF-κB is constitutively activated in many tumor cells, where it mediates cell proliferation [36,37], thus reducing susceptibility to anti-cancer drugs. In addition, mutation of *ND3* and *ND5*, which encoding the subunits of the mitochondrial respiratory chain complex I, has been reported to contribute to complex I dysfunction, changes in NADH/NAD^+^ levels, and dysregulation of the citric acid cycle [38,39,40,41,42,43]. These types of mutations affect the stability and function of mitochondrial respiration and potentially lead to changes in ROS levels [44,45,46]. The involvement of ROS in drug resistance has therefore been inferred [36,47,48,49]. In this study, we identified a T7966C mutation in *COX2* in MOLT-3 cells, a C10241Y mutation in *ND3* in MOLT-3/IDR-resistant cells, and an A12452G mutation in *ND5* in both cell lines. All mutations were located in regions encoding proteins that comprise mitochondrial complexes I and IV. Database searches revealed the *ND3* mutation site (p.Thr61Ile) to be a unique mutation that would potentially affect the activity of the mitochondrial respiratory chain complex.

Through a CGH array analysis, we next extracted five candidate genes (*GALNT2*, *DCHS2*, *ENTPD8*, *PNPLA7*, *FBXW7*) involved in drug resistance in leukemia cells (Table S1) [50,51,52,53,54,55]. The product of *GALNT2* is involved in O-glycosylation, mediating the addition of lipid moieties to carbohydrates, and protein function [56,57,58,59]. This type of post-translational modification is important for cell membrane synthesis and protein secretion. Abnormal glycosylation, a feature of most cancers, affects cell proliferation, differentiation, migration, invasion, apoptosis, and immune responses [56,60]. For this reason, *GALNT2* has been implicated in malignant control in hepatocellular carcinoma through modification of epidermal growth factor receptor (EGFR) glycosylation [61,62]. Polypeptide *N*-acetylgalactosaminyltransferases (ppGalNAc-Ts) are encoded by *GALNT* genes. Altered ppGalNAc-Ts expression has been reported to correlate with malignant transformation [63]. In addition, in human gastric cancer cells, high *GALNT2* expression is associated with remarkable inhibition of growth and metastasis [64]. In contrast, *GALNT2* has also been associated with deterioration due to human T cell leukemia [63]. Additional reports have discussed mutations of *GALNT2* in cancers [65,66]. Our sequencing analysis of *GALNT2* revealed a stop codon mutation that allowed the translation of 18 additional amino acids in the corresponding mutant protein, compared with the wild-type protein. This mutated sequence had a gene expression level of 0.35, as determined by real-time PCR. Structural prediction of the mutated *GALNT2* protein indicated changes in the binding site caused by this extended peptide sequence.

Of the candidate drug resistance genes identified in this study (*ND3* from mtDNA and *GALNT2*, *DCHS2*, *ENTPD8*, *PNPLA7*, and *FBXW7* from nuclear DNA; Table S1), a plurality of genes, including *ND3* and *GALNT2* were found to be mutated in IDR-resistant cells but not in the parental strain. Our findings suggest that multiple mutations in these genes may be related to IDR drug resistance. Future studies will analyze in greater depth the roles of these genes in IDR resistance in leukemia cells, using targeted inhibitors and cell proliferation and IDR sensitivity assays. 

## 4. Materials and Methods

### 4.1. Cell Lines and Culture Conditions

To study IDR resistance mechanisms, an IDR-resistant cell line was established from the human acute leukemia cell line MOLT-3. Specifically, MOLT-3 cells were exposed to 40 nM IDR for 8 h, after which surviving cells were subcultured without IDR for 1 week. Subsequently, the concentration of IDR was increased by 20 nM at every exposure. After 4 months, IDR resistance was tested using an MTT assay; the cells were found to be 10-fold more resistant to IDR and were designated MOLT-3/IDR (Table S4). Single colonies were subcultured and retested in an MTT assay before cryopreservation. After thawing, cells were cultured for 1 month prior to their use in subsequent experiments.

We have attempted to increase resistance in K562 and CEM cell lines to a similar level. However, we have found it difficult to increase resistance to 10-fold in these cell lines.

### 4.2. MTT Assay

MOLT-3 cells were plated in each well of a 24-well flat-bottomed plate (Corning Glass Works, Corning, NY, USA) and incubated for 3 days at 37 °C in a humidified atmosphere comprising 5% CO_2_ and 95% air. Following incubation, viable cells were quantitated using a 3-(4,5-dimethylthiazol-2-yl)-2,5-diphenyltetrazolium (MTT; Sigma, St. Louis, MO, USA) assay. Each well was treated with 50 μL of MTT solution (4 mg/mL) and 50 µL of 0.1 M sodium succinate, and plates were further incubated at 37 °C for 3 h. Next, the plates were centrifuged at 1500× *g* and 4 °C for 10 min, and the supernatants were removed. Any formazan crystals that had formed were solubilized by the addition of 750 μL of dimethylsulfoxide (DMSO), after which the contents of the wells were thoroughly mixed on a plate shaker for 5 min; subsequently, the absorbance of each well was measured at 540 nm using a spectrophotometer (Perkin Elmer, Norwalk, CT, USA) [67]. We also expressed the results as percentages of the untreated control value against the drug concentrations tested.

### 4.3. Mitochondrial DNA Sequencing

Total DNA was isolated from human T cell leukemia cell lines. MtDNA was amplified by PCR using KOD FXneo (Toyobo, Osaka, Japan). Supplemental Table S5 lists the primer sequences. The PCR amplification conditions were as follows: a denaturation step at 94 °C for 2 min, followed by 35 cycles of denaturing at 98 °C for 10 s, annealing for 20 s at 60 °C, extension at 68 °C for 1 min, and a final extension step at 68 °C for 7 min. All PCR products were fractionated by electrophoresis on 1% agarose containing Tris-borate-EDTA (TBE) at 50 V for 60 min. PCR products of the appropriate size were purified, processed by EXOSAP-IT (USB^®^ Products, Affymetrix Inc., Cleveland, OH, USA), and sequenced on an ABI 3500xL sequencer with the appropriate PCR primers. The mtDNA sequence (approximately 16,500 bp) was assembled using ATGC software (Genetyx Corporation, Tokyo, Japan).

### 4.4. CGH Array Analysis

To detect exon mutations (SNV: single nucleotide variant, SNP: single nucleotide polymorphism, STR: short tandem repeats, InDel: insertion and deletion) potentially related to IDR resistance, genomic DNA extracted from the MOLT-3 and MOLT-3/IDR cell lines was labeled with Cy3 and Cy5 dyes via dye swapping. Next, DNA was hybridized to a human genome CGH array and probed with 2 × 400 K probes covering the entire MOLT-3 chromosome. The signal intensity of each probe was detected using a microarray scanner. Probes exhibiting two-fold and four-fold increases in signal intensity in MOLT-3/IDR DNA relative to MOLT-3 DNA were extracted for further analysis. 

### 4.5. GALNT2 DNA Sequencing

Five candidate genes, *GALNT2*, *DCHS2*, *ENTPD8*, *PNPLA7*, and *FBXW7*, with potential involvement in IDR drug resistance were identified by CGH array analysis. These five gene exomes were enriched and indexed using the SureSelect XT Human All Exon 50 Mb kit (Agilent, Santa Clara, CA, USA). The samples were sequenced as 100-bp paired-end runs on a HiSeq2000 system (Illumina, San Diego, CA, USA).

Next, sequences around the stop codon of mutated *GALNT2* were examined. Total DNA was isolated from the MOLT-3 and MOLT-3/IDR cell lines and used in the following PCR mix: 5 μL of 2× KOD FX Neo Buffer, 1 µL of dNTPs (2 mM), 1 µL of each *GALNT2* primer (2.5 µM), 1.9 µL of milliQ water (Merck Millipore Corporation, Darmstadt, Germany), 0.1 µL of KOD FX Neo (Toyobo, Osaka, Japan), and 1 µL of template DNA (100 ng/µL) from MOLT-3 or MOLT-3/IDR. The PCR amplification conditions were as follows: a denaturation step at 94 °C for 2 min, followed by 35 cycles of denaturation at 98 °C for 10 s, annealing at 66 °C for 20 s, and extension at 68 °C for 20 s, and a final extension step at 68 °C for 7 min. All PCR products were fractionated by electrophoresis on 1.5% agarose gels containing TBE at 50 V for 60 min. PCR products of the appropriate sizes were purified, processed by EXOSAP-IT, and sequenced (ABI 3500xL) using PCR primers. *GALNT2* DNA sequence data were assembled using ATCG software.

### 4.6. Real-Time PCR

Real-time PCR of wild-type and mutant *GALNT2* was performed in a 10-µL reaction containing 5 µL of SYBR Premix Ex Taq II (2×) (Tli RNaseH Plus) and ROX plus (Takara Bio, Tokyo, Japan), 0.5 µL of each primer (10 µM; Table S3), and 4.5 µL of diluted template; a StepOnePlus real-time PCR system (Thermo Fisher Scientific, Foster City, CA, USA) was used for analysis. Total RNA was extracted from MOLT-3 and MOLT-3/IDR cells using TRIzol reagent (Thermo Fisher Scientific, USA), and cDNA was synthesized using the Superscript III First-Strand Synthesis System for real-time PCR (Invitrogen, Carlsbad, CA, USA). Each reaction contained 5 µg of total RNA, 20 mM Tris-HCl pH 8.4, 50 mM KCl, 2.5 μM Oligo (dT), 500 μM dNTPs, 5 mM MgCl_2_, 10 mM DTT, 2 U/µL RNase OUT (Invitrogen), and 20 U/µL Superscript III Reverse Transcriptase; reactions were incubated at 50 °C for 50 min, and 85 °C for 5 min. Next, 1 µL of RNaseH (2U) was added, followed by an incubation at 37 °C for 20 min. The cycling parameters for real-time PCR were as follows: a denaturation step at 95 °C for 20 s, followed by 45 cycles of denaturation at 95 °C for 5 s, annealing and extension for 30 s at 69 °C, 95 °C for 10 s, 60 °C for 1 min, and incremental increases up to 95 °C to produce a melting curve. Finally, real-time PCR products were fractionated by electrophoresis on 2% agarose containing TBE at 50 V for 60 min (Figure 1).

### 4.7. Reverse Transcription (RT)-PCR

RT-PCR was performed for wild-type and mutant *GALNT2* (Figure 2). The cycling parameters for PCR amplification, using ExTaq, were as follows: a denaturation step at 95 °C for 3 min, followed by 35 cycles of denaturation at 95 °C for 10 s, annealing for 10 s at 68, 69, 70, or 71 °C, extension at 72 °C for 20 s, and a final extension at 72 °C for 7 min. All PCR products were fractionated by electrophoresis on 2% agarose containing TBE at 50 V for 60 min (Figure 2). The primers are listed in Table S3.

### 4.8. Sequence Alignments of Mitochondrial DNA Sequences and GALNT2 DNA Sequences

The determined *GALNT2* and mtDNA sequences were aligned using ClustalW software. MtDNA sequences were compared to reference mtDNA from a healthy Polish individual (EU547188.2). This reference sequence harbored only four polymorphisms, compared with the human T cell leukemia cell line sequence. Mutation sites were subjected to homology searches across a range of databases (NCBI-dbSNP, MitoMap, Ensemble, JSNP), and NM.004481.3 (GI: 206725412) was chosen as the reference for *GALNT2* DNA and RNA.

### 4.9. Structural Analysis of Mutated GALNT2 Protein

A recent report presented the high-resolution X-ray structure of wild-type *GALNT2* with UDP-GalNAc [27]. In our study, a three-dimensional structural prediction for mutant *GALNT2* was developed, using wild-type *GALNT2* (PDB ID: 4D0Z) as the template structure. The X-ray structure of wild-type *GALNT2* was determined from residue 75 (Lys) up to residue 569 (Leu) of the total sequence. Therefore, in our study, our structural prediction started at residue 75 (Lys) and continued up to the extended sequence of mutant *GALNT2* (residue 569, Leu). The structural prediction for this region was performed via homology modeling, using the X-ray structure (4D0Z) as a template. Structural prediction from residue 570 to the extended sequence was performed by free modeling (i.e., not dependent on the template; Figure 3 and Figure S3). Homology modeling and free modeling were performed using Prime (Schrödinger LLC, New York, NY, USA). During modeling, the UDP-GalNAc molecule attached at 4D0Z was included in the structural prediction.

The initial structure was subjected to 100-ns molecular dynamics-based energy minimization with the Desmond program, version 3.4.0.1 [68] to ensure the structural optimization of extended sequences by free modeling. The OPLS2005 force field was used for these simulations. The initial structure was placed into TIP3P water molecules solvated with 0.15 M NaCl. After minimization and relaxation of the model, the production MD phase was performed for 100 ns in the isothermal-isobaric (NPT) ensemble at 300 K and 1 bar of pressure, using Langevin dynamics. Long-range electrostatic interactions were computed using the Smooth Particle Mesh Ewald Method. All system setups were performed using Maestro (Schrödinger LLC).

## 5. Conclusions

We aimed to elucidate the molecular mechanisms of IDR resistance in acute leukemia cells. MtDNA sequence and CGH array analyses of a human acute leukemia cell line MOLT-3 and an IDR-resistant derivative, MOLT-3/IDR, were performed. We identified a unique mutation site (p.Thr61Ile) in *ND3* from mtDNA in the MOLT-3/IDR cell line. From a CGH array analysis, we extracted five candidate drug resistance genes and focused specifically on *GALNT2*, which is associated with the O-linked glycosylation of lipids. A mutation in the stop codon of this gene led to the translation of 18 additional amino acids in the mutated protein, compared with the wild-type. The expression level of the mutated gene, as determined by real-time PCR, was 0.35. Structural analyses indicated that the extended *GALNT2* peptide sequence might alter the structure of the binding site, which could potentially affect glycosylation and lead to changes in drug resistance. Our findings offer new insight into the molecular mechanisms of IDR resistance in acute leukemia cells.

## Figures and Tables

**Table 1:** Mutation sites in the mitochondrial DNA sequences of MOLT-3/IDR cells.

Gene Name	Mitochondrial DNA Mutation Site	Region	Amino Acid Mutation Site
*COX2*	T7966C	exon	Phe127Ser
*ND3*	C10241Y	exon	Thr61Ile
*D-Loop*	16193_16194insCC	non-coding region	-

**Table 2:** Probes showing two- and four-fold increase in signal intensity on a CGH array comparison of MOLT-3/IDR versus MOLT-3 cells.

MOLT-3/IDR vs. MOLT-3	Two-Fold Signal Intensity	Four-Fold Signal Intensity
MOLT-3/IDR (MOLT-3/IDR) Duplicated	674	9
MOLT-3 (MOLT-3/IDR) Lost or highly mutated	724	1

**Table 3:** Amino acid mutation sites identified within *GALNT2* in MOLT-3 and MOLT-3/IDR cells.

Mutation Site (Nucleotide No.)	Mutation Site (Amino Acid No.)	GALNT2 Wild	MOLT-3	MOLT-3/IDR	Synonymous/Nonsynonymous
858	286	T (Threonine)	T	T	Synonymous
1014	338	I (Isoleucine)	I	I	Synonymous
1197	399	A (Alanine)	A	A	Synonymous
1716	572 (Stop codon)	X	X	Y (Tyrosine)	Nonsynonymous

**Table 4:** *GALNT2* mutation expression ratio in MOLT-3/IDR cells relative to MOLT-3 cells.

Cell Culture	GALNT2 Wild	GALNT2 Mutant
MOLT-3	1.00	N.D.(Not Detected)
MOLT-3/IDR	0.96	0.35

## Supplementary Materials

Supplementary materials can be found at www.mdpi.com/1422-0067/18/1/12/s1.
