# Supplementary material for: Analysis to Estimate Genetic Variations in the Idarubicin-Resistant Derivative MOLT-3
Source: Int J Mol Sci. 2016 Dec 22;18(1):12. doi: 10.3390/ijms18010012 (PMC5297647; doi:10.3390/ijms18010012)
Supplement: Supplementary file 1 [file ijms-18-00012-s001.pdf]

Supplementary Materials: Analysis to Estimate Genetic Variations in the Idarubicin-Resistant Derivative MOLT-3

Tomoyoshi Komiyama, Atsushi Ogura, Takatsugu Hirokawa, Miao Zhijing, Hiroshi Kamiguchi, Satomi Asai, Hayato Miyachi and Hiroyuki Kobayashi

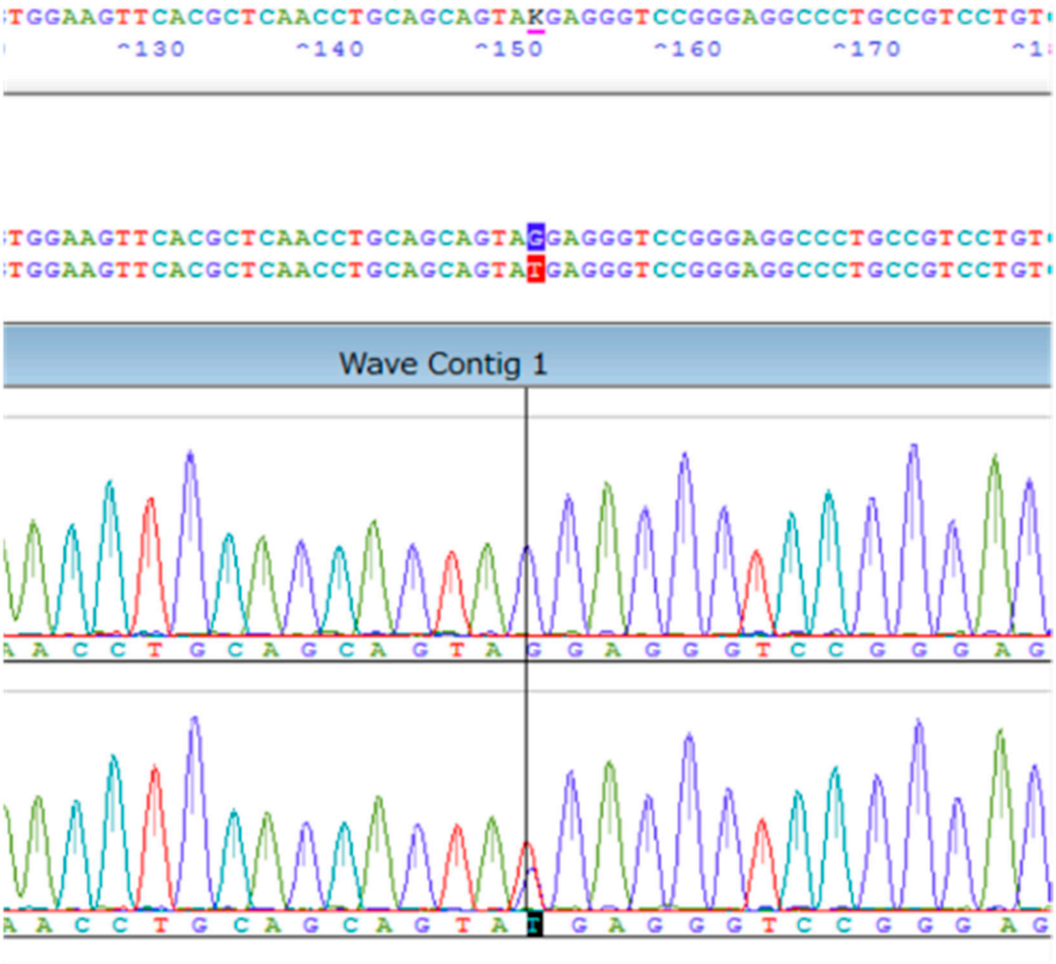

Figure S1. Sequencing profile of the stop codon mutation in the *GALNT2* gene of MOLT-3/IDR cells. Green A: Adenine, Red T: Thymine, Blue G: Guanine, Light blue C: Cytosine.

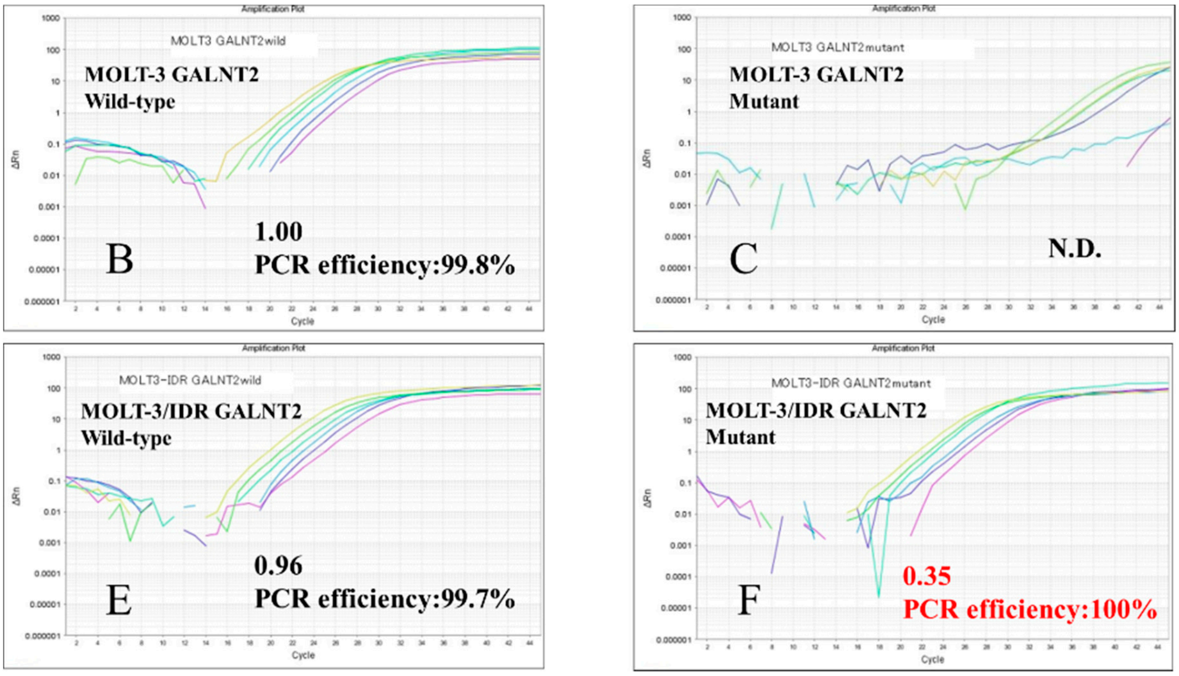

Figure S2. Gene expression quantification analysis of the *GALNT2* gene. (**B**) GALNT2 wild-type in MOLT-3 cells; (**C**) GALNT2 mutant in MOLT-3 cells; (**E**) GALNT2 wild-type of MOLT-3/IDR cells; (**F**) GALNT2 mutant in MOLT-3/IDR cells (Figure 1). N.D.: Not Detected.

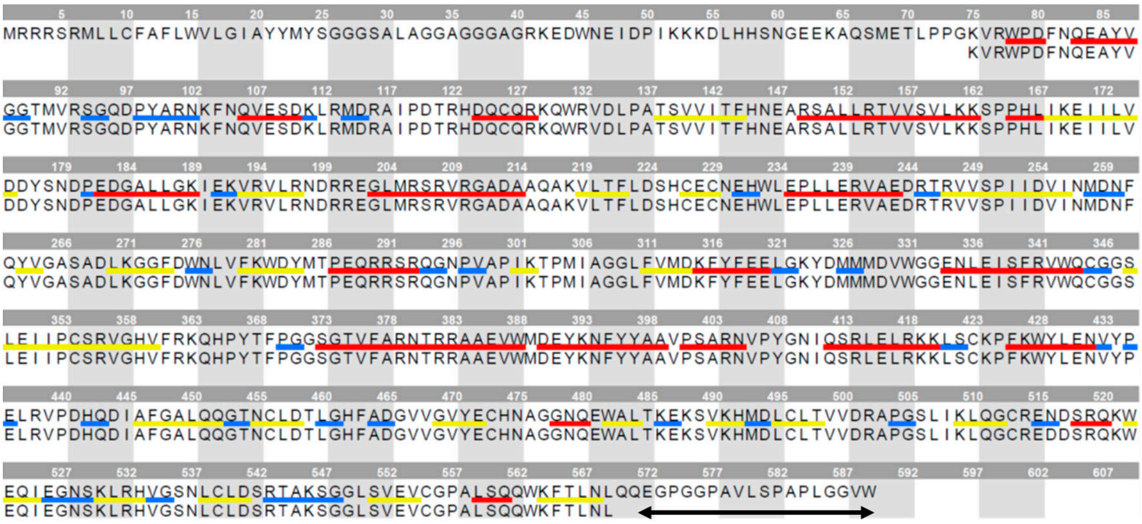

Figure S3. Alignment of the wild-type (**lower**) and mutant (**upper**) *GALNT2* protein sequences. Structure annotation of the helix, sheet, and turn structures derived from the X-ray structure of wild-type *GALNT2* are indicated by bold bars in red, yellow, and blue, respectively. The black arrow showed an additional 18 amino acids in the translated GALNT2 protein. The extended sequence region indicated by the arrow at the C-terminus is the region used as the free modeling template. The sequence alignment was created with MOE software (Chemical Computing Group).

Table S1. Four candidate genes without *GALNT2* gene from CGH array analysis.

Chrom	Left	Right	ref_seq	var_type	zygosity	var_seq1	var_seq2	Gene Name	Transcript Name	dbsnp
chr4	153247498	153247505	ttttttt	DEL	Hom	t	t	*FBXW7*	NM_001013415	
chr4	153247498	153247505	ttttttt	DEL	Hom	t	t	*FBXW7*	NM_018315	
chr4	153247498	153247505	ttttttt	DEL	Hom	t	t	*FBXW7*	NM_033632	
chr4	153249146	153249147	t	SNP	Hom	c	c	*FBXW7*	NM_001013415	rs4419460
chr4	153249146	153249147	t	SNP	Hom	c	c	*FBXW7*	NM_018315	rs4419460
chr4	153249146	153249147	t	SNP	Hom	c	c	*FBXW7*	NM_033632	rs4419460
chr4	153258886	153258887	a	SNP	Het	g	a	*FBXW7*	NM_001013415	
chr4	153258886	153258887	a	SNP	Het	g	a	*FBXW7*	NM_018315	
chr4	153258886	153258887	a	SNP	Het	g	a	*FBXW7*	NM_033632	
chr4	153267968	153267969	a	SNP	Hom	g	g	*FBXW7*	NM_001013415	rs2714803
chr4	153267968	153267969	a	SNP	Hom	g	g	*FBXW7*	NM_018315	rs2714803
chr4	153267968	153267969	a	SNP	Hom	g	g	*FBXW7*	NM_033632	rs2714803
chr4	153268238	153268242	aaaa	DEL	Het	a	aaaa	*FBXW7*	NM_001013415	
chr4	153268238	153268242	aaaa	DEL	Het	a	aaaa	*FBXW7*	NM_018315	
chr4	153268238	153268242	aaaa	DEL	Het	a	aaaa	*FBXW7*	NM_033632	
chr4	153329073	153329074	t	SNP	Het	c	t	*FBXW7*	NM_033632	
chr4	155156207	155156208	g	SNP	Hom	a	a	*DCHS2*	NM_017639	rs7655799
chr4	155157041	155157042	g	SNP	Het	t	g	*DCHS2*	NM_017639	
chr4	155161666	155161667	c	SNP	Hom	t	t	*DCHS2*	NM_017639	rs6535989
chr4	155180652	155180653	c	SNP	Het	a	c	*DCHS2*	NM_017639	rs111516993
chr4	155191710	155191712	gt	MNP	Het	aa	gt	*DCHS2*	NM_017639	
chr4	155219318	155219319	c	SNP	Het	t	c	*DCHS2*	NM_017639	rs28561984
chr4	155219361	155219362	a	SNP	Het	g	a	*DCHS2*	NM_017639	rs17031387
chr4	155219662	155219663	g	SNP	Het	c	g	*DCHS2*	NM_017639	rs17031394
chr4	155225879	155225880	c	SNP	Het	t	c	*DCHS2*	NM_017639	
chr4	155226421	155226422	c	SNP	Het	a	c	*DCHS2*	NM_017639	rs12507663
chr4	155236778	155236779	c	SNP	Hom	g	g	*DCHS2*	NM_017639	rs1388085

Table S1. *Cont.*

Chrom	Left	Right	ref_seq	var_type	zygosity	var_seq1	var_seq2	Gene Name	Transcript Name	dbsnp
chr4	155241438	155241439	g	SNP	Het	a	g	*DCHS2*	NM_017639	rs11935503
chr4	155241572	155241573	g	SNP	Het	a	g	*DCHS2*	NM_017639	rs11935573
chr4	155243573	155243574	c	SNP	Hom	t	t	*DCHS2*	NM_017639	rs13109747
chr4	155243604	155243605	t	SNP	Hom	c	c	*DCHS2*	NM_017639	rs1352714
chr4	155244475	155244476	g	SNP	Hom	t	t	*DCHS2*	NM_001142552	rs12500437
chr4	155244475	155244476	g	SNP	Hom	t	t	*DCHS2*	NM_017639	rs12500437
chr4	155244499	155244502	aaa	DEL	Het		aaa	*DCHS2*	NM_001142552	
chr4	155244499	155244502	aaa	DEL	Het		aaa	*DCHS2*	NM_017639	
chr4	155249431	155249432	t	SNP	Het	c	t	*DCHS2*	NM_001142552	rs1472070
chr4	155249431	155249432	t	SNP	Het	c	t	*DCHS2*	NM_017639	rs1472070
chr4	155256177	155256178	a	SNP	Het	g	a	*DCHS2*	NM_001142552	rs6858157
chr4	155256177	155256178	a	SNP	Het	g	a	*DCHS2*	NM_017639	rs6858157
chr4	155256205	155256206	g	SNP	Hom	a	a	*DCHS2*	NM_001142552	rs6858712
chr4	155256205	155256206	g	SNP	Hom	a	a	*DCHS2*	NM_017639	rs6858712
chr4	155264637	155264638	g	SNP	Het	a	g	*DCHS2*	NM_001142552	rs62331892
chr4	155264637	155264638	g	SNP	Het	a	g	*DCHS2*	NM_017639	rs62331892
chr4	155295168	155295171	aaa	DEL	Het		aaa	*DCHS2*	NM_001142552	
chr4	155295168	155295171	aaa	DEL	Het		aaa	*DCHS2*	NM_017639	
chr4	155295171	155295178	aaaaaaa	DEL	Hom			*DCHS2*	NM_001142552	
chr4	155295171	155295178	aaaaaaa	DEL	Hom			*DCHS2*	NM_017639	
chr4	155305491	155305492	c	SNP	Het	t	c	*DCHS2*	NM_001142552	rs34927139
chr4	155305491	155305492	c	SNP	Het	t	c	*DCHS2*	NM_017639	rs34927139
chr4	155312411	155312412	g	SNP	Het	a	g	*DCHS2*	NM_001142552	rs62331900
chr4	155312411	155312412	g	SNP	Het	a	g	*DCHS2*	NM_017639	rs62331900
chr4	155407407	155407408	a	SNP	Hom	t	t	*DCHS2*	NM_001142552	rs4346631
chr4	155407569	155407570	t	SNP	Hom	c	c	*DCHS2*	NM_001142552	rs12501998

Table S1. *Cont.*

Chrom	Left	Right	ref_seq	var_type	zygosity	var_seq1	var_seq2	Gene Name	Transcript Name	dbsnp
chr4	155407595	155407596	c	SNP	Het	a	c	*DCHS2*	NM_001142552	
chr4	155407595	155407596	c	SNP	Het	a	c	*DCHS2*	NM_001142553	
chr4	155407680	155407681	a	SNP	Hom	g	g	*DCHS2*	NM_001142552	rs13151559
chr4	155407680	155407681	a	SNP	Hom	g	g	*DCHS2*	NM_001142553	rs13151559
chr4	155410284	155410285	a	DEL	Hom			*DCHS2*	NM_001142552	rs34485270
chr4	155410284	155410285	a	DEL	Hom			*DCHS2*	NM_001142553	rs34485270
chr4	155410407	155410408	c	SNP	Het	g	c	*DCHS2*	NM_001142552	rs9999285
chr4	155410407	155410408	c	SNP	Het	g	c	*DCHS2*	NM_001142553	rs9999285
chr4	155410822	155410823	g	SNP	Hom	a	a	*DCHS2*	NM_001142552	rs4696593
chr4	155410822	155410823	g	SNP	Hom	a	a	*DCHS2*	NM_001142553	rs4696593
chr4	155411065	155411066	t	SNP	Hom	c	c	*DCHS2*	NM_001142552	rs7656522
chr4	155411065	155411066	t	SNP	Hom	c	c	*DCHS2*	NM_001142553	rs7656522
chr4	155411104	155411105	a	SNP	Hom	c	c	*DCHS2*	NM_001142552	rs6536016
chr4	155411104	155411105	a	SNP	Hom	c	c	*DCHS2*	NM_001142553	rs6536016
chr9	140328878	140328879	t	SNP	Het	g	t	*ENTPD8*	NM_001033113	
chr9	140328878	140328879	t	SNP	Het	g	t	*ENTPD8*	NM_198585	
chr9	140329618	140329619	a	SNP	Het	g	a	*ENTPD8*	NM_001033113	rs28589413
chr9	140329618	140329619	a	SNP	Het	g	a	*ENTPD8*	NM_198585	rs28589413
chr9	140330976	140330977	t	SNP	Het	c	t	*ENTPD8*	NM_001033113	rs13297080
chr9	140330976	140330977	t	SNP	Het	c	t	*ENTPD8*	NM_198585	rs13297080
chr9	140331128	140331129	t	SNP	Het	c	t	*ENTPD8*	NM_001033113	
chr9	140331128	140331129	t	SNP	Het	c	t	*ENTPD8*	NM_198585	
chr9	140331833	140331834	a	SNP	Hom	g	g	*ENTPD8*	NM_001033113	rs9414731
chr9	140331833	140331834	a	SNP	Hom	g	g	*ENTPD8*	NM_198585	rs9414731
chr9	140332478	140332479	a	SNP	Het	g	a	*ENTPD8*	NM_001033113	rs6606582
chr9	140332478	140332479	a	SNP	Het	g	a	*ENTPD8*	NM_198585	rs6606582

Table S1. *Cont.*

Chrom	Left	Right	ref_seq	var_type	zygosity	var_seq1	var_seq2	Gene Name	Transcript Name	dbsnp
chr9	140332668	140332669	c	SNP	Het	t	c	*ENTPD8*	NM_001033113	
chr9	140332668	140332669	c	SNP	Het	t	c	*ENTPD8*	NM_198585	
chr9	140356110	140356111	a	SNP	Het	c	a	*PNPLA7*	NM_001098537	
chr9	140356110	140356111	a	SNP	Het	c	a	*PNPLA7*	NM_152286	
chr9	140356374	140356375	c	SNP	Hom	a	a	*PNPLA7*	NM_001098537	rs4962240
chr9	140356374	140356375	c	SNP	Hom	a	a	*PNPLA7*	NM_152286	rs4962240
chr9	140356503	140356504	c	SNP	Het	a	c	*PNPLA7*	NM_001098537	rs41307436
chr9	140356503	140356504	c	SNP	Het	a	c	*PNPLA7*	NM_152286	rs41307436
chr9	140356807	140356808	g	SNP	Het	t	g	*PNPLA7*	NM_001098537	
chr9	140356807	140356808	g	SNP	Het	t	g	*PNPLA7*	NM_152286	
chr9	140357972	140357973	a	SNP	Hom	g	g	*PNPLA7*	NM_001098537	rs1891627
chr9	140357972	140357973	a	SNP	Hom	g	g	*PNPLA7*	NM_152286	rs1891627
chr9	140358121	140358122	c	SNP	Het	t	c	*PNPLA7*	NM_001098537	
chr9	140358121	140358122	c	SNP	Het	t	c	*PNPLA7*	NM_152286	
chr9	140361847	140361848	g	SNP	Het	a	g	*PNPLA7*	NM_001098537	rs61738892
chr9	140361847	140361848	g	SNP	Het	a	g	*PNPLA7*	NM_152286	rs61738892
chr9	140373556	140373557	g	SNP	Het	a	g	*PNPLA7*	NM_001098537	
chr9	140373556	140373557	g	SNP	Het	a	g	*PNPLA7*	NM_152286	
chr9	140373562	140373563	g	SNP	Het	a	g	*PNPLA7*	NM_001098537	
chr9	140373562	140373563	g	SNP	Het	a	g	*PNPLA7*	NM_152286	
chr9	140374861	140374862	a	SNP	Hom	g	g	*PNPLA7*	NM_001098537	rs1891630
chr9	140374861	140374862	a	SNP	Hom	g	g	*PNPLA7*	NM_152286	rs1891630
chr9	140378978	140378979	a	SNP	Het	g	a	*PNPLA7*	NM_001098537	
chr9	140378978	140378979	a	SNP	Het	g	a	*PNPLA7*	NM_152286	
chr9	140392494	140392495	t	SNP	Het	c	t	*PNPLA7*	NM_001098537	rs4962232

Table S1. *Cont.*

Chrom	Left	Right	ref_seq	var_type	zygosity	var_seq1	var_seq2	Gene Name	Transcript Name	dbsnp
chr9	140392494	140392495	t	SNP	Het	c	t	*PNPLA7*	NM_152286	rs4962232
chr9	140395119	140395120	g	SNP	Het	a	g	*PNPLA7*	NM_001098537	
chr9	140395119	140395120	g	SNP	Het	a	g	*PNPLA7*	NM_152286	
chr9	140396006	140396007	c	SNP	Het	t	c	*PNPLA7*	NM_001098537	
chr9	140396006	140396007	c	SNP	Het	t	c	*PNPLA7*	NM_152286	
chr9	140396087	140396088	c	DEL	Het		c	*PNPLA7*	NM_001098537	
chr9	140396087	140396088	c	DEL	Het		c	*PNPLA7*	NM_152286	
chr9	140396212	140396213	c	SNP	Het	t	c	*PNPLA7*	NM_001098537	rs1891629
chr9	140396212	140396213	c	SNP	Het	t	c	*PNPLA7*	NM_152286	rs1891629
chr9	140399983	140399984	t	SNP	Het	c	t	*PNPLA7*	NM_001098537	rs7037200
chr9	140399983	140399984	t	SNP	Het	c	t	*PNPLA7*	NM_152286	rs7037200
chr9	140400275	140400276	t	SNP	Het	c	t	*PNPLA7*	NM_001098537	rs3750381
chr9	140400275	140400276	t	SNP	Het	c	t	*PNPLA7*	NM_152286	rs3750381
chr9	140400361	140400362	g	SNP	Het	a	g	*PNPLA7*	NM_001098537	
chr9	140400361	140400362	g	SNP	Het	a	g	*PNPLA7*	NM_152286	
chr9	140403817	140403818	a	SNP	Het	g	a	*PNPLA7*	NM_001098537	rs34204672
chr9	140403817	140403818	a	SNP	Het	g	a	*PNPLA7*	NM_152286	rs34204672
chr9	140403835	140403836	c	SNP	Het	t	c	*PNPLA7*	NM_001098537	rs35605405
chr9	140403835	140403836	c	SNP	Het	t	c	*PNPLA7*	NM_152286	rs35605405
chr9	140409726	140409727	t	SNP	Hom	c	c	*PNPLA7*	NM_001098537	rs843967
chr9	140409726	140409727	t	SNP	Hom	c	c	*PNPLA7*	NM_152286	rs843967
chr9	140440242	140440243	c	SNP	Het	t	c	*PNPLA7*	NM_001098537	
chr9	140440242	140440243	c	SNP	Het	t	c	*PNPLA7*	NM_152286	

Table S2. Level of resistance to IDR in MOLT-3 and MOLT-3/IDR cells.

**MOLT-3**	**β ACTIN**	**GALNT2 Wild**	**GALNT2 Mutant**
Approximation	$y = -3.3187x + 18.752$	$y = -3.3574x + 24.627$	
	$R^2 = 0.9998$	$R^2 = 0.9986$	
PCR efficiency	1.001348901	0.985406898	
**MOLT-3/IDR**	**β ACTIN**	**GALNT2 Wild**	**GALNT2 Mutant**
Approximation	$y = -3.2816x + 17.893$	$y = -3.3292x + 23.829$	$y = -3.2951x + 25.301$
	$R^2 = 0.9956$	$R^2 = 0.9899$	$R^2 = 0.9999$
PCR efficiency	1.017109154	0.996974236	1.011318861
Threshold Cycle (C_t)			
	β ACTIN	GALNT2 wild	GALNT2 mutant
MOLT-3	18.752	24.627	
MOLT-3/IDR	17.893	23.829	25.301
Δ Threshold Cycle (Target C_t)—(βACTIN C_t)			
	GALNT2 wild	GALNT2 mutant	
MOLT-3	5.875		
MOLT-3/IDR	5.936	7.408	
ΔΔ Threshold Cycle (MOLT-3/IDR ΔC_t)—(MOLT-3 ΔC_t)			
	GALNT2 wild	GALNT2 mutant	
MOLT-3	0		
MOLT-3/IDR	0.061(=5.936–5.875)	1.533 (=7.408–5.875)	
**Quantitative Expression Analysis Using Real-Time PCR of the *GALNT2* Gene**			
	GALNT2 wild	GALNT2 mutant	
MOLT-3	1	N.D. (Not Detected)	
MOLT-3/IDR	0.96	0.35	

Table S3. Primers used for real-time PCR and RT-PCR.

**Primer Name**	**Real-Time PCR Primer Sequence**
GALNT2-wild_F	5′-CGCTCAACCTGCAGCAGTAG-3′
GALNT2-mutant_F	5′-ACGCTCAACCTGCAGCAGTAT-3′
GALNT2-Rev	5′-CTTGCCGAAGTTTGACTTTCAG-3′
**Primer Name**	**RT-PCR Primer Sequence**
GALNT2-L1	5′-CCTAAGCGTGGAGGTGTGTG-3′
GALNT2-L2	5′-CGCTCAACCTGCAGCAGTAT-3′
GALNT2-Rev	5′-CTTGCCGAAGTTTGACTTTCAG-3′

Table S4. Level of resistance to IDR in MOLT-3 and MOLT-3/IDR cells.

**Drug Name**	**MOLT-3**	**MOLT-3/ IDR**	**Degree of Resistance**
Idarubicin	25 nM	250 nM	10-fold

Table S5. PCR primers used to amplify mtDNA sequences.

Primer Name	Forward/Reverse	Primer Sequence
homoF1	F	5′-AGGACTATTCCTAGCCATGCACTACTCA-3′
homoR1	R	5′-AAATAATAGGATGAGGCAGGAATCAAAGAC-3′
homoF2	F	5′-GAACTGTATCCGACATCTGGTTCCTACT-3′
homoR2	R	5′-TTTAGCTCAGAGCGGTCAAGTTAAGTTG-3′
homoF3	F	5′-TATGAAACTTAAGGGTCGAAGGTGGATT-3′
homoR3	R	5′-GCCATCTTAACAAACCCTGTTCTTG-3′
homoF4	F	5′-ATCCAATAACTTGACCAACGGAACA-3′
homoR4	R	5′-AAGGATTATGGATGCGGTTGCTT-3′
homoF5	F	5′-CCACCTATCACACCCCATCCTAA-3′
homoR5	R	5′-CAGTTGCCAAAGCCTCCGATT-3′
homoF6	F	5′-CAATGCTTCACTCAGCCATTTTACCT-3′
homoR6	R	5′-TGTAGACCTACTTGCGCTGCATGT-3′
homoF7	F	5′-GATGCATACACCACATGAAACATCCTA-3′
homoR7	R	5′-GTGATTGGTGGGTCATTATGTGTTGT-3′
homoF8	F	5′-ACTTCTTACCACAAGGCACACCTACA-3′
homoR8	R	5′-GGCAATAGGCACAATATTGGCTAAGA-3′
homoF9	F	5′-GATAATCATATTTACCAAATGCCCCTCATT-3′
homoR9	R	5′-GCAGTTCTTGTGAGCTTTCTCGGTAA-3′
homoF10	F	5′-CACTCACCCACCACATTAACAACA-3′
homoR10	R	5′-GGCTGTTAGAAGTCCTAGGAAAGTGACAG-3′
homoF11	F	5′-GCATTAGCAGGAATACCTTTCCTCACA-3′
homoR11	R	5′-CAAGAATAGGAGGTGGAGTGCTGCTA-3′
Homo_mt_Gap_1F	F	5′-GGCCACCAATGGTACTGAAC-3′
Homo_mt_Gap_1R	R	5′-GCAATGAATGAAGCGAACAG-3′
Homo_mt_Gap_2F	F	5′-TTAGACTGAGCCGAATTGGT-3′
Homo_mt_Gap_2R	R	5′-AGCCTCTGTTGTCAGATCCA-3′
Homo_mt_Gap_3F	F	5′-TCGGAGGACAACCAGTAAGC-3′
Homo_mt_Gap_3R	R	5′-GCACTCTTGTGCGGGATATT-3′
HUMmt-148F	F	5′-ATCCCATTATTTATCGCACCT-3′
HUMmt-1038R	R	5′-CTATTGTGTGTTCAGATATGTT-3′
HUMmt-1038F	F	5′-ACATATCTGAACACACAATAGCT-3′
HUMmt-1914F	R	5′-AGACGAGCTACCTAAGAACAGCT-3′
HUMmt-2022R	F	5′-AACTAAGATTCTATCTTGGACA-3′
HUMmt-2850R	R	5′-TGGTGAAGTCTTAGCATGTACT-3′
HUMmt-4885F	F	5′-ATCTCAATCATATACCAAATCT-3′
HUMmt-5606R	R	5′-ATTTGCGTTCAGTTGATGCAGA-3′
HUMmt-13601F	F	5′-TATAGCACTCGAATAATTCTT-3′
HUMmt-14133F	F	5′-ACTCCTAATCACATAACCTATT-3′
HUMmt-14201R	R	5′-TGATTAGTAGTAGTTACTGGTT-3′
HUMmt-15052R	R	5′-AGTAGAGAAATGATCCGTAATA-3′
F817-837	F	5′-GGGAAACAGCAGTGATTAACC-3′
R1235-1255	R	5′-CAAGAGGTGGTGAGGTTGATC-3′
F1557-1579	F	5′-GAGGAGACAAGTCGTAACATGGT-3′
R2341-2360	R	5′-ATCTGACGCAGGCTTATGC-3′
F2592-2613	F	5′-CGTGCAAAGGTAGCATAATCAC-3′
R3071-3091	R	5′-ACTCCGGTCTGAACTCAGATC-3′
F5536-5556	F	5′-ACAGACCAAGAGCCTTCAAAG-3′
R6015-6032	R	5′-AGCTCGGCTCGAATAAGG-3′
F6818-6838	F	5′-CATATTTCACCTCCGCTACCA-3′
R7419-7440	R	5′-GTATACGGGTTCTTCGAATGTG-3′
